# Quantitative Estimation of Itopride Hydrochloride and Rabeprazole Sodium from Capsule Formulation

**DOI:** 10.4103/0250-474X.45411

**Published:** 2008

**Authors:** S. Pillai, I. Singhvi

**Affiliations:** Pacific College of Pharmacy, Pratap Nagar Extension, Airport Road, Udaipur-313 001, India

**Keywords:** Spectrophotmetric, HPLC, itopride hydrochloride and rabeprazole sodium

## Abstract

Two simple, accurate, economical and reproducible UV spectrophotometric methods and one HPLC method for simultaneous estimation of two component drug mixture of itopride hydrochloride and rabeprazole sodium from combined capsule dosage form have been developed. First developed method involves formation and solving of simultaneous equations using 265.2 nm and 290.8 nm as two wavelengths. Second method is based on two wavelength calculation, wavelengths selected for estimation of itopride hydrochloride was 278.0 nm and 298.8 nm and for rabeprazole sodium 253.6 nm and 275.2 nm. Developed HPLC method is a reverse phase chromatographic method using phenomenex C_18_ column and acetonitrile: phosphate buffer (35:65 v/v) pH 7.0 as mobile phase. All developed methods obey Beer's law in concentration range employed for respective methods. Results of analysis were validated statistically and by recovery studies.

Itopride hydrochloride chemically, N-[4-[2-(dimethylamino)ethoxy]-benzyl]-3,4-dimethoxy-benzamide hydrochloride^1^ is a gastroprokinetic agent^2^. Literature survey reveals that for itopride hydrochloride HPLC^3^–^4^ methods have been reported. Rabeprazole sodium, chemically 2-[[[4-(3-methoxypropoxy)-3-methyl-2-pyridinyl]methyl]sulfinyl]-1H-benzimidazole sodium is the latest proton pump inhibitor and is used in the management of acid related disorders^5^. Few analytical methods for estimation of rabeprazole sodium from biological fluid including HPLC^6^,^7^, LC-MS^8^, LC-NMR^9^, column switching LC^10^ and spectrophotometric^11^–^12^ are reported. However no spectrophotometric or HPLC method is yet reported for simultaneous analysis of two drugs from combined pharmaceutical dosage form.

A Systronics UV/Vis double beam spectrophotometer (model 2101) with 1 cm matched quartz cells was used for spectrophotometric analysis. Spectra were recorded using specific program of instrument, having specifications as, spectral band width 2 nm, wavelength accuracy ± 0.5 nm, wavelength readability 0.1 nm increment. For HPLC method Shimadzu LC-10AT with SPD-10A detector was used. Different batches of the capsule samples of combined dosage form of itopride hydrochloride and rabeprazole sodium [Itza RB (Cadila Pharmaceutical Pvt. Ltd, Ahmadabad)] were procured from the local market.

In the first method, pure drug sample of itopride hydrochloride and rabeprazole sodium were dissolved separately in distilled water so as to give eight dilutions of standard in concentration range of 5-40 μg/ml of itopride hydrochloride and 2 - 30 μg/ml of rabeprazole sodium. All solutions were scanned in wavelength range of 220.0 nm and 380.0 nm. Fig. 1 represents the overlain spectra of itopride hydrochloride and rabeprazole sodium in distilled water. Two wavelengths selected for formation and solving of simultaneous equations were 265.2 nm and 290.8 nm. Absorptivity coefficients of both the drugs were determined at selected wavelengths. Absorptivity coefficient for itopride hydrochloride at 265.2 nm and 290.8 nm were 338.64 and 168.50 cm^−1^ g^−1^ l while respective values for rabeprazole sodium were 231.35 and 320.65 cm^−1^ g^−1^ l. Set of two simultaneous equations thus formed are, A_1_= 338.64C_1_+231.35C_2_---I and A_2_=168.5C_1_+320.65C_2_----II, where A_1_ and A_2_ are absorbance of sample solution at 265.2 nm and 290.8 nm, respectively. C_1_ and C_2_ are concentration of itopride hydrochloride and rabeprazole sodium respectively in sample solution in g/l. Validity of above formed equations was checked by preparing five mixed standards using pure drug sample of two drugs, results of which are reported in Table 1.

**Fig. 1:**
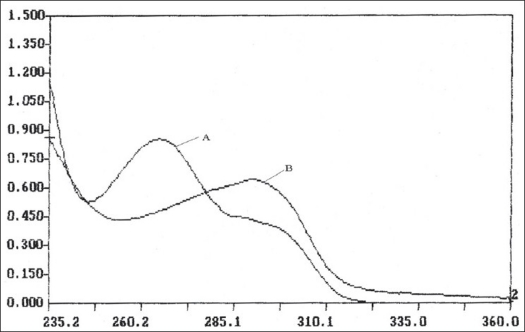
Overlain spectra of itopride hydrochloride and rabeprazole sodium A: Spectra of itopride hydrochloride in distilled water. B: Spectra of rabeprazole sodium in distilled water.

**TABLE 1:** RESULTS OF VALIDATION STUDIES FOR METHOD I AND II USING MIXED STANDARDS

Sample No.	Conc. Present (mcg/ml)		% Conc. Found			
	
			Method I		Method II	
			
	ITP	RAB	ITP	RAB	ITP	RAB
01	30	04	99.96	98.75	101.36	98.44
02	25	08	101.60	102.12	101.25	98.34
03	20	12	100.38	100.30	98.16	96.90
04	15	16	101.72	100.29	100.71	98.34
05	10	20	99.60	98.94	99.78	99.36

ITP represents itopride hydrochloride and RAB denotes rabeprazole sodium

For analysis of formulation contents of twenty capsules were accurately weighed and average weight per capsule was determined, contents were grounded to fine powder and powder equivalent to 150 mg of itopride hydrochloride was accurately weighed and extracted four times with 20 ml portions of distilled water and filtered through Whatman filter paper No. 41 into a 100 ml volumetric flask and volume was made up to the mark with the same. From the above filtrate 5 ml was further diluted to 50 ml with distilled water in a volumetric flask. Finally 2 ml was diluted to 10 ml in a volumetric flask. The absorbance of this final diluted sample solution was measured at 265.2 nm and 290.8 nm respectively and concentration of two drugs in the sample were calculated using above framed simultaneous Eqns. I and II. Results of analysis of capsule formulation are reported in Table 2.

**TABLE 2:** RESULTS OF ANALYSIS OF COMMERCIAL FORMULATION

Method	Batch	Label claim (mg/cap)		% of label claim estimated*		Standard Deviation		% Recovery**	
					
		ITP	RAB	ITP	RAB	ITP	RAB	ITP	RAB
	A	150	20	99.66	99.75	0.428	0.462	100.53	99.66
Method I	B	150	20	99.44	99.52	0.514	0.522	100.94	100.75
	C	150	20	99.01	98.98	0.562	0.554	100.27	100.20
	A	150	20	98.92	99.12	0.659	0.425	99.52	99.54
Method II	B	150	20	99.21	99.54	0.588	0.408	100.12	99.11
	C	150	20	99.60	99.22	0.482	0.398	99.66	98.91
Method III	A	150	20	100.30	100.88	0.251	0.605	100.77	99.94
	B	150	20	100.15	99.50	0.468	0.582	99.92	99.33
	C	150	20	99.81	99.47	0.424	0.561	99.95	99.66

ITP represents itopride hydrochloride and RAB denotes rabeprazole sodium.

* Average of three determinations.

** Average of determination at three different concentration levels. Batch A, B and C: Three different batches of Itza RB

In the second method, based on the absorption spectra of itopride hydrochloride and rabeprazole sodium (fig. 1), a set of two wavelengths λ_1_ (278.0 nm) and λ_2_ (298.8 nm) for estimation of itopride hydrochloride and λ_3_ (253.6 nm) and λ_4_ (275.2 nm) for estimation of rabeprazole sodium were selected on basis of principle that absorbance difference between two points on a mixture spectra is directly proportional to concentration of component of interest and independent of interfering component. Five mixed standard of pure drug containing different concentration of two drugs were prepared in distilled water. All standards were scanned at respective set of selected wavelengths. Absorbance difference was measured and respective calibration curve was plotted. Capsule sample solution was prepared in similar manner as for the first method and final sample solution was analyzed by scanning at respective set of wavelength and determined absorbance difference values were noted, the concentration of itopride hydrochloride and rabeprazole sodium was calculated from the respective calibration curve. Result of analysis is reported in Table 2.

A high performance liquid chromatographic method was also developed using phenomenex C_18_ ODS (5 μ) 250×4.60 mm column, mobile phase selected for this method contains 65 parts of phosphate buffer (0.1 M, 13.6 g of KH_2_PO_4_ in 1000 ml distilled water) and 35 parts of acetonitrile, adjusted to pH 7.0 with triethyl amine which was filtered through 0.2 μ membrane filter. Flow rate employed was 1.0 ml/min. Detection of eluent was carried out at 276.0 nm. Pure drug sample of itopride hydrochloride and rabeprazole sodium were dissolved separately in mobile phase so as to give a standard stock of 100 μg/ml for each drug. For preparation of drug solutions for the calibration curve, in a series of 10 ml volumetric flask seven dilutions of 5-60 μg/ml of itopride hydrochloride and 2-50 μg/ml of rabeprazole sodium were prepared. Each solution was injected and a chromatogram was recorded. Mean retention time for itopride hydrochloride was found to be 4.22 min and for rabeprazole sodium 8.76 min. The peak area of itopride hydrochloride and rabeprazole sodium was calculated and respective calibration curves were plotted against concentration of drug and peak area.

For analysis of capsule formulation contents of twenty capsules were accurately weighed and average weight per capsule was determined. Contents were grounded to fine powder and powder equivalent to 150 mg of itopride hydrochloride was accurately weighed and transferred to a 100 ml volumetric flask and about 75 ml of mobile phase was added. The powder mixture was dissolved in the mobile phase with the aid of ultrasonication. The solution was filtered through Whatman filter paper No. 41 into another 100 ml volumetric flask washed the filter paper with mobile phase and added washings to the filtrate. Volume of the filtrate was made up to the mark with the mobile phase. From the above filtrate 5 ml was further diluted to 50 ml with mobile phase in a volumetric flask. Finally 2 ml was diluted to 10 ml with mobile phase in a volumetric flask, this solution was filtered through 0.2 μ membrane filter.

After setting the chromatographic conditions and stabilizing the instrument, the capsule sample solution was injected and a chromatogram was recorded. The injection was repeated three times and the peak areas were recorded. A representative chromatogram has been given in fig. 2. The peak area for each drug was calculated and the amount of each drug present per capsule was estimated from the respective calibration curve. Results of analysis of capsule formulation are reported in Table 2.

**Fig 2:**
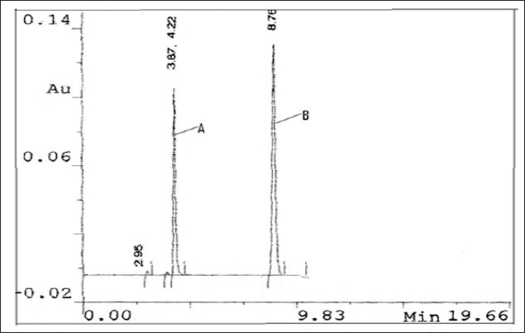
Chromatogram for itopride hydrochloride and rabeprazole Sodium A: Represents chromatogram of itopride hydrochloride in mobile phase while. B: Denotes chromatogram of rabeprazole sodium in mobile phase

To study the accuracy, reproducibility and precision of the above developed methods recovery studies were carried out by addition 0.5, 1.0 and 1.5 ml of standard drug stock solution (100 μg/ml) of each drug to pre-analyzed capsule sample solutions. Results of recovery studies were found to be satisfactory and are reported in Table 2.

Two spectrophotometric and one HPLC methods have been developed for simultaneous estimation of itopride hydrochloride and rabeprazole sodium from combined capsule dosage form. The first developed method involving formation and solving of simultaneous equations is very simple and requires only accurately determined absorptivity of the two drugs at two selected wavelengths. The method just requires recording of absorbances and few calculations that can be manually done, thus method can be used with any model of spectrophotometer. Once the equations are framed the method is very fast. Framed equations were validated using laboratory prepared mixed standards of two drugs which gave satisfactory results.

Second developed method for simultaneous analysis of itopride hydrochloride and rabeprazole sodium makes use of two wavelength calculation so as to remove interference between two components. Proper selection of two wavelengths for estimation of a component is critical. The third developed method for simultaneous estimation of two drugs from combined dosage form is reverse phase chromatographic method utilizing C_18_ column and acetonitrile: phosphate buffer as mobile phase. Detection of eluent was carried out using UV detector. The run time per sample is just 12 min.

The result of analysis of two drugs from of capsule formulation using these developed methods were found close to 100% for both itopride hydrochloride and rabeprazole sodium, values of standard deviation was satisfactorily low indicating accuracy and reproducibility of the methods. Recovery studies were satisfactory which shows that there is no interference of excipients. The developed methods were found to be simple, rapid, accurate and can be used for routine analysis of two drugs from combined capsule formulations.
